# Ether as an Anæsthetic Agent.—Hypnotism

**Published:** 1860-02

**Authors:** 


### EDITORIAL DEPARTMENT.

*Ether as an Anæsthetic Agent.—Hypnotism.*—The more anæsthesia is employed in surgery, and the wider its application becomes in practical medicine and obstetrics, the more do we realize the terrible results which may arise from the administration of chloroform, and the immense superiority of ether as an anæsthetic agent, as far as regards the safety of the patient. We are gratified to see that this subject is attracting more and more the attention of the profession. We now hardly think a surgeon has a right to administer chloroform, when so many cases of death are being reported every day, and these in the most skillful and experienced hands. Of all agents for producing insensibility, we believe that ether is the most free from danger, and if pure, it can almost be said that it is absolutely safe.

As a general rule, the lower animals are more susceptible to the influence of anæsthetics than the human subject; and all who have had occasion to use these agents upon them to any extent must be convinced that chloroform is exceedingly unsafe. So much so, indeed, that we are about as likely to kill the animal with chloroform, even when given with the greatest care, as to administer it successfully. We have had occasion to operate this winter, however, upon dogs and cats, always putting them under the influence of ether, and have been surprised several times by the unexpected death of the animal. This has been entirely unexpected, for we have been able to administer ether with the greatest freedom, and, indeed, have found it difficult to kill an animal with it, even when we desired to do so. This circumstance we have explained by the impurity of the ether; and we think that we can learn an important lesson from this in our administration of ether to the human subject. But one or two cases of death from the administration of ether in the human subject, are upon record. One of them was presented by Dr. Alonzo Clark, to the Pathological Society some months ago, and was published with the Proceedings in this Journal. In this case, our readers will remember, post-mortem examination disclosed disease of the cerebellum, which made it not a fair case of death from the effects of the ether. Though deaths from ether so seldom occur, we see by the effects of impure ether upon the lower animals, that a bad article is much more dangerous than a good one, and that it is of the greatest importance that this article should be perfectly pure. It has long been held that death from chloroform only takes place as a result of the impurity of the agent; and its advocates have contended that when pure it was perfectly safe. That the former is not the case, has now been abundantly proven; though an impure article is much more dangerous than the pure. Sufficient attention has not, we think, been paid to the quality of the ether used for anæsthetic purposes; a point, which, we are convinced should not be overlooked.

Among the other novelties which now agitate the medical world, is a new mode of producing insensibility, called by the French *l'anesthesie hypnotique.*

A young French surgeon, Dr. Axam, recently went to Paris for the purpose of communicating to the scientific bodies of that city this method of producing anæsthesia. He presented the result of his observations to MM. Broca, Follin, and others, and with them made a series of experiments. This, however, was not an entirely new discovery, but has been known as far back as 1842, when it was mentioned in an English work; still, it has, till now, been entirely forgotten, and has all the novelty of a new discovery. Anæsthesia is produced by Dr. Axam in the following manner:

"The subject is seated or reclined in a convenient position; the operator, placed before or behind him, fixes before his eyes, a few inches distant, but generally short of the point of distinct vision, a shining body, on which the eyes are to be directed and fixed in a steady manner; the shining body should be placed in such a manner, that the eyes, in order to see it, must be directed strongly upwards by the superior recti muscles contracted to their fullest extent. In that attitude there is a forced contraction of the elevators of the eye-lids and the superior recti; and also convergent strabismus.

"Hardly has this attitude, sufficiently fatiguing indeed, been prolonged for two or three minutes, when we see the pupils contract, then dilate, the eye-lids tremble rapidly and close; and soon the patient is unconscious; then two symptoms, almost constant, manifest themselves, but they are more or less marked, and of greater or less duration. They are: First, catalepsy, in every way similar to that which is described in classic works; Second, anæsthesia, which lasts from three to fifteen minutes, which is complete or incomplete, but which ordinarily permits of forcible pinching, pricking, or tickling of the patient, without the slightest trace of sensibility appearing, or without even those external irritations mollifying in any way the cataleptic state.

"This state of anæthesia is ordinarily followed by a period of an entirely opposite state, characterized by a condition of very remarkable hyperæsthesia, in which we see the ordinary sensibility, the sensations of temperature and of muscular activity attain a degree of impressibility entirely unusual. At all periods of the experiment we are able to quickly abolish these symptoms by making, as M. Puel has already observed in his researches on catalepsy, frictions over the eyelids, or in directing a brisk current of cold air over these organs.

"The subjects, when they have returned to themselves, do not retain any recollections of the preceding moments."*

This is an account of the procedure by which this wonderful hypnotism is produced. Experiments have been made upon this subject by many distinguished surgeons of Paris, and some painful operations have been performed while patients were in the state described, without the slightest consciousness or recollection on their part. Other experiments have failed, and it is found that all are not amenable to the influence of hypnotism. Individuals of the male sex have been brought under its influence as well as females. Though it is by no means certain of how much use the application of this principle may be, it demands, indorsed as it has been abroad, a fair trial, which of course can be easily made by any one.

* Gazette Hebdomadaire, 9 Décembre, 1859.

